# Vitamin B12 deficiency in an infant secondary to nutritional deficiency and an inadequate maternal diet

**DOI:** 10.1002/jpr3.70200

**Published:** 2026-07-16

**Authors:** Sandra Sala‐Lluch, Nerea Mesas‐Vila, Susana Ferrando‐Monleón, Ana Barrés‐Fernández, Cristina Villar‐Vera, José Vicente Arcos‐Machancoses

**Affiliations:** ^1^ Department of Pediatrics Hospital Clínic Universitari de València Valencia Spain; ^2^ Universitat de València València Spain; ^3^ INCLIVA Biomedical Research Institute Valencia Spain; ^4^ Pediatric Gastroenterology Hepatology and Nutrition Department Hospital Clínic Universitari de València Valencia Spain

**Keywords:** breastfeeding, developmental delay, micronutrient deficiency

## Abstract

Vitamin B12 (cobalamin, Cbl) is an essential micronutrient for DNA synthesis and neurological development. Its deficiency in infants, although infrequent in developed countries, can cause megaloblastic anemia, psychomotor delay, and neurological damage that may become irreversible if not treated early. We present the case of a 16‐month‐old infant, breastfed with partial refusal of solid foods, whose mother followed a diet poor in animal products and had low levels of Cbl in breast milk. The patient consulted for fever and somnolence, with clinical findings of hypotonia, global developmental delay, and pancytopenia. Laboratory workup confirmed severe Cbl deficiency with hyperhomocysteinemia and elevated methylmalonic acid. Congenital and malabsorptive causes were excluded. Intramuscular Cbl therapy led to rapid hematological and neurological improvement, together with nutritional support adapted due to cow's milk protein allergy. This case highlights the importance of early detection and adequate supplementation in at‐risk mothers and infants.

## 1 INTRODUCTION

Vitamin B12 or cobalamin (Cbl) is an essential micronutrient for cellular growth and central nervous system development. It is obtained from animal‐derived foods.^1^

In Europe, Cbl deficiency in children is uncommon but can occur mainly in infants born to breast‐fed by B12‐deficient mothers, especially those following vegetarian or vegan diets and, less frequently, malabsorption disorders. Epidemiological studies conducted in Europe have reported data on Cbl deficiency. In Germany, the annual incidence of Cbl deficiency among newborns is estimated at 1 in 9600, while the incidence among symptomatic infants under 1 year of age is approximately 1 in 17,500.^2^ Additionally, a study carried out in Norway found out that 8% of infants aged 6–15 months present Cbl deficiency, which was observed more frequently among breastfed infants.^1^

It is considerably more prevalent in Asia and Africa due to low intake of animal‐source foods influenced by cultural, religious, and socioeconomic factors.^3^

Cbl functions as a coenzyme for methionine synthase, which converts homocysteine (Hcy) into methionine for protein synthesis and DNA methylation, and for the mitochondrial enzyme methylmalonyl‐CoA mutase, which isomerizes methylmalonyl‐CoA into succinyl‐CoA to feed the citric acid cycle. An excess of methylmalonyl‐CoA results in raised levels of methylmalonic acid (MMA). Lack of Cbl inactivates these enzymes, forcing the accumulation of Hcy and MMA. High levels of these metabolites are directly neurotoxic, causing the oxidative stress and abnormal fatty acid synthesis that ultimately lead to demyelination of nerve fibers.^4^ Clinical manifestations range from megaloblastic anemia and pancytopenia to neurological impairment, including developmental delay. In infants, gastrointestinal symptoms such as vomiting, feeding difficulties, and growth delay may also be present. Early diagnosis is essential, as prolonged deficiency can lead to irreversible neurological damage.^1^

The present case is relevant due to the rarity of this condition in developed countries and its association with breastfeeding in the context of maternal diet poor in animal‐based foods and concurrent malnutrition.

## 2 CASE REPORT

We present a 16‐month‐old infant, who was born at term after a normal pregnancy. He is the third child of non‐consanguineous parents. The mother was previously healthy but followed a diet poor in animal products (only one or two servings per week) for religious reasons. The patient continued breastfeeding and followed a diet mainly based on vegetables and greens, with minimal animal products. Since the introduction of solid foods, the patient had experienced intermittent vomiting and partial refusal of solids, which had not previously been evaluated.

He consulted to the emergency department with somnolence and fever of 24‐h duration. On examination, the infant showed pallor, dehydration, fatigue, hypotonia, hyperpigmentation of knuckles, sparse, and depigmented hair. Growth parameters were within normal limits. However, developmental assessment revealed global delay, particularly in gross motor skills, with inability to sit, crawl, or walk independently and poor postural control. Cognitive and sociocommunicative delays were also evident. Notably, psychomotor development had been considered normal 3 months earlier during routine pediatric follow‐up.

Initial analysis showed pancytopenia, megaloblastic anemia, and reticulocytopenia. Peripheral smear confirmed pancytopenia and hypersegmented neutrophils. Serum Cbl levels were <109 pmol/L (normal values > 258 pmol/L) with normal folate.

Further workup showed elevated Hcy, 17.56 μmol/L (normal value < 10 μmol/L), and urinary MMA 66 mmol/mol creatinine (normal value 0.2–8.5 mmol/mol creatinine). Electroencephalography and brain magnetic resonance imaging were normal.

Maternal evaluation revealed serum Cbl of 185 pmol/L, suggesting a possible vitamin B12 deficiency, with hyperhomocysteinemia 15.38 μmol/L (normal value < 10 μmol/L). Because maternal serum levels do not reliably predict breast milk cobalamin content, breast milk analysis was performed and demonstrated low Cbl concentration 109 pmol/L (normal value > 226 pmol/L).

Inherited disorders of cobalamin metabolism and malabsorption syndromes such as Imerslund–Gräsbeck disease were excluded by metabolic testing and genetic analysis.

Fever, which was the initial reason for consultation, was attributed to influenza A infection, confirmed by polymerase chain reaction testing.

During refeeding, the patient developed anaphylaxis to a high‐calorie polymeric formula. This required a switch to an elemental formula and exclusion of cow's milk protein (CMP), later confirmed by elevated casein‐specific immunoglobulin E, which likely explained the longstanding vomiting and feeding intolerance.

Measures to prevent refeeding syndrome were implemented, including correction of electrolyte abnormalities, thiamine supplementation, careful fluid management, and gradual nutritional advancement.

Treatment was initiated with intramuscular cyanocobalamin at a dose of 1.000 µg/72 h (three doses in total), following established recommendations.^5^ The parenteral route was selected because of neurological symptoms and concerns adherence in the context of vomiting. Clinical improvement was observed within the first week, with progressive hematological recovery and marked improvement in motor function. Given the rapid response, only the loading phase of treatment was required. He was discharged after 3 weeks, fully tolerating complementary feeding while continuing a CMP–free diet and without the need of cyanocobalamin supplementation. At 1‐month follow‐up, Cbl (578 pmol/L) and Hcy (8 μmol/L) levels had normalized.

## 3 DISCUSSION

Cbl deficiency in exclusively breastfed infants is mainly related to inadequate maternal intake. A higher risk of deficiency has been linked to women following vegan or vegetarian diets. However, evidence shows that approximately 69% of omnivorous mothers with low intake of animal products do not meet recommended Cbl requirements,^6^ which are set by the *European Food Safety Authority (EFSA)* at 4.5 µg/day during pregnancy and 5 µg/day during lactation.^4^ Maternal Cbl deficiency may be asymptomatic, but it can have critical consequences for the infant, particularly during the first 6 months of life, when breast milk is the main source of Cbl.^6^

Studies have also reported that mothers receiving oral Cbl supplementation may still present low plasma levels and elevated serum Hcy, highlighting the importance of monitoring markers of Cbl status during pregnancy and lactation.^6^

Several sources have highlighted the correlation between maternal and infant Cbl levels, with a critical maternal serum threshold <300 pmol/L associated with elevated infant MMA.^7^ To date, the cutoff of <310 pmol/L has been used as a reference for low Cbl in human milk; however, the recent Mothers, Infants, and Lactation Quality (MILQ) study reports a concentration of <226 pmol/L.^8^ Although serum Cbl measurement is widely used, it has limitations due to false positives and lack of consensus regarding cutoff values. MMA is considered the gold standard biomarker, while holotranscobalamin offers greater diagnostic accuracy,^6^ although the possibility of performing this laboratory test is limited in most clinical settings, as in this case.

Regarding treatment, both intramuscular and high‐dose oral Cbl have been shown to be effective in pediatric deficiency even in malabsorption syndromes,^9^ but higher concentrations are likely more easily achieved through parenteral supplementation.^10^

## 4 CONCLUSION

In this case, clinical and biochemical response to intramuscular therapy was fast and sustained. Nutritional etiology was confirmed, and severe congenital defects were excluded. Identification of concomitant CMP allergy influenced dietary management, underscoring the complexity of nutritional care in this patient.

## CONFLICT OF INTEREST STATEMENT

The authors declare no conflicts of interest.

## ETHICS STATEMENT

Written informed consent was obtained from the parents of the patient for the publication of this case report.
